# An Account of the Poisonous Quality of the Juice of the Root of Jatropha Manihot, or Bitter Cassada; and of the Use of Cayenne Pepper in Counteracting the Effects of This and Some Other Poisonous Substances; with Remarks on the Efficacy of the Spigelia Anthelmia in Worm Cases

**Published:** 1797

**Authors:** James Clark

**Affiliations:** Physician in Dominica, and Fellow of the College of Physicians and Royal Society of Edinburgh.


XXV. *An Account of the poisonous Quality of the Juice of the Root of Jatropha Manihot, or bitter Cassada; and of the Use of Cayenne Pepper in counteracting the Effects of this and some other poisonous Substances; with Remarks on the Efficacy of the Spigelia Anthelmia in Worm Cases. Communicated in a Letter to Dr.* Simmons, *by* James Clark, *M.D. Physician in Dominica, and Fellow of the College of Physicians and Royal Society of Edinburgh.*

THE pernicious quality of the juice of the roots of *Jatropha Manihot*, or bitter Cassada, has been known ever since these islands were first cultivated by Europeans. It is not certain whether this plant is indigenous, or whether it has been brought from Surinam, Demerary, or

ſome other part of South America, where it is planted, prepared, and uſed as bread by the Indians, in the ſame manner as it is by the red Caraibs, or true Aborigines of theſe iſlands, at this time.

From the very ſudden effects of the juice of the roots of this plant on negroes who had drank it intentionally, or on thoſe who had eaten the roots by miſtake; and alſo from the fatal and ſpeedy effects of it on horſes, mules, cattle, goats, ſheep, and on all kinds of poultry, which I have been an eye witneſs of, I have been induced to conſider it as the moſt powerful narcotic vegetable poiſon that we are acquainted with at preſent, not excepting even the cherry laurel water. The Indians of South America, and the Caraibs of theſe iſlands, who appear to be the ſame race of men, boil this juice with Cayenne pepper and ſalt, which they uſe as a ſauce to their fiſh, and ſoak their Caſſada bread in it before eating it; from which circumſtance I was led to make a few experiments, in order to aſcertain whether boiling deſtroyed the poiſonous quality of this juice, or whether its effects as a narcotic were counteracted or deſtroyed by the pepper.

In July, 1794, I procured four large bull frogs, frogs *(Rana latrans,)* and after grating ſeveral roots of Caſſada, and preſſing out the juice, I put half a pint of it apart, and boiled a pint of it ſlowly, till it was reduced one half, of which, after letting it cool, I gave four tea ſpoonfuls to two frogs, and the ſame quantity of the raw juice to two more; not more than one half remained in the ſtomachs of each. Three hours after, the two frogs that had ſwallowed the raw juice died. I repeated the doſe of the boiled juice to the two others at this time, and one of them died in an hour after, but the other ſurvived ſome hours; it was, however, motionleſs, and inſenſible to the prick of a pin at the time the other died. From this I found that the boiled juice poiſoned as well as the raw, although not quite ſo ſoon. But ſuſpecting that the juice had not been boiled ſufficiently, I had ſome more boiled down to one fourth of the quantity, when it became a little thick, reſembling a thin jelly of ſtarch. I gave four tea ſpoonfuls of this juice to two frogs as before, and the ſame quantity of raw juice to two more. One of theſe that took the boiled juice died in three hours, and the other in the night, after having had the doſe repeated; as did thoſe which had taken the raw juice: it might be about five hours

U 2 from from the time the juice was given to each of them, that they died. It would feem from thefe trials, that the boiled juice poifoned fooner than the raw; but upon repeating thefe experiments frequently, it was found, in general, that the raw juice was more quickly fatal. The difference in this inftance arofe probably from the whole quantity of the juice not reaching the ftomach, or from their having rejected a great part of it after it had been fwallowed. This happened, more or lefs, to be the cafe in all the experiments upon them.

As thefe animals are known to be remarkably tenacious of life, I thought it might give fome idea of the great power of this poifon, to know how long they would live after having been deprived of their vital parts. With a view to this object, I cut out the heart of a frog, and ftitched up the thorax immediately, and at the fame time I cut off the head of another frog; the heart continued to beat an hour, and both frogs lived near two hours; fo that they were killed nearly as foon by the Caffada water, as they were by deftroying parts fo effential to life.

I poifoned lizards alfo with this juice, but the effects of it upon animals with cold blood, was not near fo fudden as on thofe with warm blood, fuch as fheep, kids, turkies, fowls, &c.

I have known a ftrong negro die in little more than an hour after drinking perhaps half a pint or more of this juice; and a ftrong mule in much lefs time. Negroes who had eaten the roots roafted, lived three or four hours after. Finding, from thefe trials, that neither boiling nor roafting deftroyed the poifonous quality of the Caffada root, I fufpected that the capficum, or Cayenne pepper, boiled in it by the Caraibs had this effect, and that it might be the real antidote for it. I therefore procured more frogs, to fome of which I gave the raw juice and the boiled, as in the former experiments, and with the fame effect; to others I gave the juice boiled with a fufficient quantity of capficum to make it tafte pretty hot. The frogs that took the juice in this way lived, and feemed to be enlivened by it every time it was repeated, which was very frequently, and they did not die afterwards.

The white people in South America ufe this fauce with their fifh, as the Caraibs do in all the iflands where they refide, without fuffering any inconvenience or bad confequence from it. It appears, therefore, that the action of the capficum, as a powerful ſtimulant to the ſtomach, and to the ſyſtem in general, counteracts the ſedative or narcotic powers of this juice. Capſicum has been known, long ago, to poſſeſs the power of counteracting or preventing the poiſonous effects of fiſh; and ſtrong liquors, wine, and ſpices are adminiſtered with the ſame intention to thoſe who happen to be affected by poiſonous fiſh. This fiſh poiſon ſeldom deſtroys life entirely, except the deadly poiſon of the yellow bill'd ſprat, as it is called, which kills very ſpeedily; but thoſe who have eaten of the other kinds of poiſonous fiſh, are frequently reduced to the laſt extremity by the vomiting, and life is almoſt extinguiſhed before ſtimulants can take effect.

A ſingular effect of fiſh poiſon is to remove the epidermis in patches, or ſpots, about the hands and feet, which continue white in people of colour, and of a pale yellow colour in white people, for life.

The cauſe of ſome kinds of fiſh being poiſonous, I ſuſpect to be their feeding on ſubmarine narcotic weeds, and not to their feeding on copperas banks in the ſea, as hath been commonly ſuppoſed. The effects of the poiſon of the black land crab, which feeds upon the mountain mountain Mahault, are exactly ſimilar to thoſe ariſing from poiſonous fiſh.

The treatment of thoſe who have taken a quantity of the Caſſada juice, or eaten the root, is the ſame as is generally practiſed for all other narcotic poiſons, viz. to empty the ſtomach as ſpeedily as poſſible, and afterwards to adminiſter the moſt powerful cordials and ſtimulants.

In caſes of this ſort, when I have been called before the patient had loſt the power of ſwallowing, I have always given, to full grown robuſt people, half a drachm or two ſcruples of white vitriol, and leſs in proportion to ſuch as were weakly, or to children. This doſe commonly operated in ten minutes, and ſometimes ſooner, if the patient had not become inſenſible, in which caſe the doſe was repeated ſoon after. In every caſe I have ſeen, there was ſuch a violent ſpaſmodic contraction of the muſcles of the throat, that it was with the utmoſt difficulty any liquid could be got down. I have ſeen none die, however, who could ſwallow.

After the operation of a vomit, ſtrong ginger tea with ſome rum in it, when wine could not be procured, was given in ſmall quantities at a time, till the comatoſe ſymptoms (which proved conly fatal when no internal remedy could be administered) were entirely removed. Some have been carried off by a ſtrong convulſion; and large evacuations by ſtool were the forerunner of death. The ſtomach and bowels were always diſtended to their utmoſt extent, and the pupils of the eyes were much dilated.

I have opened the bodies of many who died of this poiſon, but could diſcover nothing remarkable, only that a great deal of very offenſive air ruſhed from the ſtomach when it was opened, and the Caſſada, or part of the juice was found in the bottom of it.

No caſes of negroes being poiſoned by this juice, have occurred to me ſince I found the capſicum to be an antidote for it.

Cayenne pepper grows in great plenty upon all eſtates in this iſland, and on ſuch as are at a diſtance from medical aid, and not provided with a vomit to adminiſter directly, it ought to be tried. A quantity of it bruiſed in warm water and poured into the ſtomachs of horſes, ſheep, poultry, or ſtock of any kind, which have drank this juice, might be the means of ſaving them. No means hitherto tried for the recovery of animals that have drank it, have proved ſucceſsful. Capſicum, would, no doubt, relieve relieve thoſe who have eaten *farine* (or the root) not perfectly dried, by which the bowels are ſo much diſtended, as ſometimes to endanger life.

When I was employed in making theſe trials on Caſſada water, I alſo poiſoned frogs, lizards, &c. with the juice of ſome other narcotic plants which are indigenous here, viz.

*Solanum mammoſum*, Cock-roach Apple.

*Jatropha Curcas*, Engliſh Phyſic Nut.

——— *urens*, Prickly Nut.

*Datura ferox*, A ſpecies of Thorn Apple.

*Spigelia Anthelmia*, Worm Graſs, a ſpecies of Indian Pink, called, by the French Inhabitants, *Brenvillié*.

The juice of the four firſt proved fatal to the frogs nearly as ſoon as that of the Caſſada; but it is extremely acrimonious and therefore difficult to be given. The laſt is not ſo powerful a narcotic, and has been long in uſe as an anthelmintic. It differs from the *Spigelia Marilandica*, or Indian pink, deſcribed by the late Dr. Garden, of Charles Town, South Carolina, in the third volume of Eſſays Phyſical and Literary; the roots of the *Spigelia Anthelmia*, being ſo fibrous, that it cannot be reduced to powder; but the plant plant poſſeſſes the ſame virtues in its leaves, ſeeds, and ſtalks, that the other ſpecies, or Indian pink, does in its roots.

The leaves boiled, or infuſed like tea, form a very powerful vermifuge, but when given in too large a doſe, it has proved fatal to very young children, and it has on this account been laid aſide for ſome time. Of the leaves and ſeeds, dried and pounded, from five to ten grains may be given, however, to a child above two years old without any riſk; but the moſt common way of preparing this remedy for worms, is to make a ſtrong infuſion of the whole plant in boiling water; to which, a quantity of the rinds of four oranges, or lemons, is added, and ſome of the juice alſo; this is afterwards ſtrained, and boiled to the conſiſtence of ſyrup*, with muſcovado ſugar. A table ſpoonful or two of this ſyrup given to a child from two to ſix years of age, thrice a day, for two days running, and a proper doſe of *Ol. Ricini*, the

* A handful of the plant to a gallon of water is the uſual quantity. This is allowed to infuſe, or rather ſimmer, for twenty-four hours, till one-fourth part of it has evaporated. The orange peel and acid are added, and a ſufficient quantity of ſugar to form it into a ſyrup, about an hour before it is taken off the fire.

day after, feldom fail to bring away a number of *lumbrici*, or round worms, to which children, particularly in warm climates, are remarkably fubject. It is feldom given to children under two years old; and during its ufe, it is neceffary to confine the patients in a dark room, as the light makes them quite giddy, the pupils of their eyes being dilated in an aftonifhing manner, and they feel a pain in the balls of their eyes. When given in too large dofes it occafions dizzinefs, lofs of fight, and flight comatofe fymptoms, which are removed by a fpoonful or two of lime juice and water, or vinegar, and by wafhing the face with cold water. It feldom occafions ficknefs at the ftomach; and when ufed in fmall dofes, and the precautions above mentioned had been attended to, I have never known a fingle inftance of its proving fatal, or even giving occafion for a ferious alarm. It is rarely adminiftered, however, until other vermifuge medicines have been tried without fuccefs, or until the cafe becomes very urgent and dangerous, owing to prejudices entertained againft it. The number of worms that are difcharged by the ufe of this fyrup, in the fpace of two or three days, is almoft incredible; they often amount to fifty or fixty, and fometimes to a hundred hundred in that time. The ſyrup might be ſent to England, but it would not keep long enough to be equally efficacious there. The dried leaves and ſeeds, however, might be ſent and uſed in powder; in which ſtate I have no doubt of its proving as powerful and ſafe a vermifuge, as it does in the form of ſyrup.

*Dominica,*

*May* 1, 1796.

